# Annotations

**Published:** 1898-05-07

**Authors:** 


### ANNOTATIONS.

### Underfed Children.

CERTAIN articles in the *Times* have drawn attention to our underfed children, and to the hardships involved in sending them to schools with empty stomachs. All that has been said is absolutely true, and every charitable instinct seems to urge that at all hazards these hungry children should be fed. Yet we are faced by the apparently insoluble problem how on the one hand to feed these little starvelings, and on the other how to avoid such lightening of parental responsibility as might lead merely to the production of so many more mouths to feed. We are not quite satisfied that those who in the name of political economy would harden their hearts, and would throw all the responsibility for their children's upbringing upon the parents are altogether in the right. The "population question," so-called, is a terrible bugbear to some people. But, after all, notwithstanding our growing numbers, wealth also is increasing, so that we may take it that each good worker both keeps himself and does a little more. It is, then, of far more importance to us as a nation that each child that grows up should be turned into a good worker than that the number of children should be kept down, and more especially is this the case when we consider that the way suggested for keeping down the population involves the starving of multitudes of children during their time of growth, and thus the production of ravenous crowds of feeble, unproductive workers. By all means let us recover from neglectful parents all that it is possible to recover of what is spent upon their children; by all means let us adopt every method to deter people from recklessly crowding the world with children whom they cannot or will not bring up properly; but when the children once are there let it be remembered, as a guidance to State policy, that the condition of the country twenty years hence will largely depend upon whether those children turn out good workers, each adding strength to the social fabric, or hungry wastrels—parasites, each one of whom will be a source of weakness to the State.

### Hybrids.

IN the current number of *Science Progress* a very full account is given by F. A. Dixey of recent investigations regarding hybridisation which tend to throw some light upon various dark places in the subject of evolution. True as it probably is that new species result from the special development of pre-existing organs in response to special conditions of environment, and from a process of mutual selection by individuals best fitted to carry on the race, and easy as it is to conceive of the gradual evolution of new species under such slowly but continually acting influences, the phenomena of hybridisation are far too interesting to be overlooked, and one need not be surprised to find people ready to regard this process as being responsible for the origin of some at least of the many species which at present people the world. That hybrids are usually infertile, and that varieties produced by crossing tend to die out may be admitted, but the rule does not seem to be absolute. Again, although it is true that hybrids when they do breed tend to revert, and that when a male hybrid breeds with a normal female the offspring of this "back crossing" is of the type of the female parent, yet it is by no means certain that in such cases the reversion is complete, or that the hybrid strain may not have impressed some sort of influence upon the race. One of the most interesting points in Mr. Dixey's paper has to do with the very different results which occur when a species is crossed on the one hand with local races of the same species and on the other with accidental "sports" or aberration forms. It appears that when the normal form is crossed with a sporadic aberration the result is in many cases that the issue divides itself sharply between the normal form and the sport, some being like one and some like the other, but intermediate forms being absent. This is seen in the well-known case of tortoiseshell, tabby, and black cats. These domestic varieties exist side by side in the same race, and even in the same litter, and true intermediates are rare or absent, which would suggest that they originally appeared as sports. When, however, the normal form is crossed with a local race which is being gradually established by the accumulation of slight changes, the tendency is to the production of a series of intermediate forms, and the same is the case in crossing with a distinct species. For the production, then, of a fusion of characteristics, such as one would expect in a new species derived from hybridisation, one must look to crossing with a distinct species, or with a very gradually formed race, and not to crossing with a sport, for sports produce mixed litters rather than new and intermediate species.

### The Reception-House in St. George's, Southwark.

THE reception-house lately provided by the vestry of St. George's, Southwark, has not been long in proving its utility. A case of typhus fever having occurred, the four families inhabiting the small house where the disease had appeared were at once removed into the reception-house, while the infected house was being cleaned. The overcrowding which is so constantly found as an antecedent of outbreaks of typhus fever was well illustrated in this case, the four families in the house in question having been lodged in five small rooms. In all, ten persons were lodged and bathed, and their clothes disinfected by steam, while the house was being made safe. In the outbreak from which this case was derived it is believed that a man, his wife, seven children, the medical attendant, and the undertaker were attacked with the disease. At first there was some doubt as to the nature of the cases, but three of the sufferers died. There can be no question as to the immense advantage of having a proper reception-house to which the dwellers in infected houses can be removed on the first outbreak of the disease, and much credit is due to Dr. Waldo for his persistence in urging his vestry to provide such a refuge. We cannot say, however, that the vestry deserves all the praise which they have received from some quarters for their action in the matter. They ought never to have required any urging, for what they have done is only a statutory duty clearly imposed upon them by Act of Parliament. Strangely enough, they are rather pluming themselves just now.

